# Exploring Virtual Worlds for Scenario-Based Repeated Team Training of Cardiopulmonary Resuscitation in Medical Students

**DOI:** 10.2196/jmir.1426

**Published:** 2010-09-03

**Authors:** Johan Creutzfeldt, Leif Hedman, Christopher Medin, Wm. LeRoy Heinrichs, Li Felländer-Tsai

**Affiliations:** ^5^Stanford University Medical Media and Information TechnologyStanford UniversityPalo AltoUnited States; ^4^Department of Biomedical EngineeringKarolinska University HospitalStockholmSweden; ^3^Department of PsychologyUmeå UniversityUmeåSweden; ^2^Center for Advanced Medical SimulationKarolinska Institutet and Karolinska University HospitalStockholmSweden; ^1^Department of Clinical Science, Intervention and TechnologyKarolinska InstitutetStockholmSweden

**Keywords:** Cardiopulmonary resuscitation, concentration, educational technology, medical students, MMVW, avatars, patient simulation, self-efficacy

## Abstract

**Background:**

Contemporary learning technologies, such as massively multiplayer virtual worlds (MMVW), create new means for teaching and training. However, knowledge about the effectiveness of such training is incomplete, and there are no data regarding how students experience it. Cardiopulmonary resuscitation (CPR) is a field within medicine in high demand for new and effective training modalities.

**Objective:**

In addition to finding a feasible way to implement CPR training, our aim was to investigate how a serious game setting in a virtual world using avatars would influence medical students’ subjective experiences as well as their retention of knowledge.

**Methods:**

An MMVW was refined and used in a study to train 12 medical students in CPR in 3-person teams in a repeated fashion 6 months apart. An exit questionnaire solicited reflections over their experiences. As the subjects trained in 4 CPR scenarios, measurements of self-efficacy, concentration, and mental strain were made in addition to measuring knowledge. Engagement modes and coping strategies were also studied. Parametric and nonparametric statistical analyses were carried out according to distribution of the data.

**Results:**

The majority of the subjects reported that they had enjoyed the training, had found it to be suitable, and had learned something new, although several asked for more difficult and complex scenarios as well as a richer virtual environment. The mean values for knowledge dropped during the 6 months from 8.0/10 to 6.25/10 (*P* = .002). Self-efficacy increased from before to after each of the two training sessions, from 5.9/7 to 6.5/7 (*P* = .01) after the first and from 6.0/7 to 6.7/7 (*P* = .03) after the second. The mean perceived concentration value increased from 54.2/100 to 66.6/100 (*P* = .006), and in general the mental strain was found to be low to moderate (mean = 2.6/10).

**Conclusions:**

Using scenario-based virtual world team training with avatars to train medical students in multi-person CPR was feasible and showed promising results. Although we found no evidence of stimulated recall of CPR procedures in our test-retest study, the subjects were enthusiastic and reported increased concentration during the training. We also found that subjects’ self-efficacy had increased after the training. Despite the need for further studies, these findings imply several possible uses of MMVW technology for future emergency medical training.

## Introduction

Medical simulation is an effective instrument for training of technical as well as nontechnical skills [1-3]. Training in virtual reality, hosted online and accessed by personal computers, is an emerging learning technology that offers new possibilities. Serious games are intended to provide an engaging and self-reinforcing context in which to motivate and educate the users [4,5]. Multi-user computer environments make it possible to interact with other players in a massively multiplayer virtual world (MMVW) [6]. Heinrichs and coworkers have found MMVW training to be promising in the area of emergency medicine for repeated practice of uncommon, life-threatening trauma cases [7]; however, as Hansen points out in a review of the subject, more research concerning training outcomes is needed [8]. Further, the users’ experiences of leisure gaming technology for explicit medical training purposes should be investigated.

We have used the teaching of cardiopulmonary resuscitation (CPR) as a model for a scenario-based MMVW training approach. For more than 40 years, attention has been focused on disseminating knowledge and skills in the field of CPR. It is believed that with a better-trained population, many lives could be saved by means of bystander CPR [9,10]. Concern also exists about the quality of current CPR training because of issues such as poor retention, lack of training in so-called nontechnical skills, and negative reactions to the training [11-17].

In this study, we explored initial effects on students using a virtual world for CPR training [18]. No systematic studies regarding the effect of stimulated recall on retention of knowledge and nontechnical skills have been reported. We concur with Kneebone [19], who has identified self-efficacy as an important feature of successful simulations in terms of learning and clinical outcomes, and we also suggest concentration and mental strain as important variables of learning and performance to be analyzed for the first time in CPR training [20-25]. We hypothesized that repeated team training of CPR in a virtual world would influence self-efficacy, concentration, and mental strain in medical students as well as enhance retention of knowledge.

## Methods

We enrolled 12 medical students attending the first year at Karolinska Institutet in a test-retest explorative study. The medical students participated voluntarily as study subjects. The students’ experiences of the training were investigated using a questionnaire. Our effect measures of training were knowledge, self-efficacy, concentration, and mental strain. We controlled for engagement modes and coping strategies since students might relate differently to the virtual world as well as cope with events during training in different ways. These might in turn affect their performance and other subjective reactions differently during the two study sessions.

A test-retest design created an opportunity to follow retention and temporal changes of training effects. Based upon results from previous CPR studies [26], a 6-month interval between sessions was chosen. In order to control for expected differences between men and women both regarding computer interests and gaming preferences, we enrolled 50% of each sex. The study was approved by the regional ethics committee at Karolinska Institutet. Before entering the study, the subjects had received traditional CPR training, (bystander CPR [27]) during the previous 3 months as a component of their regular medical school curriculum.

**Figure 1:**
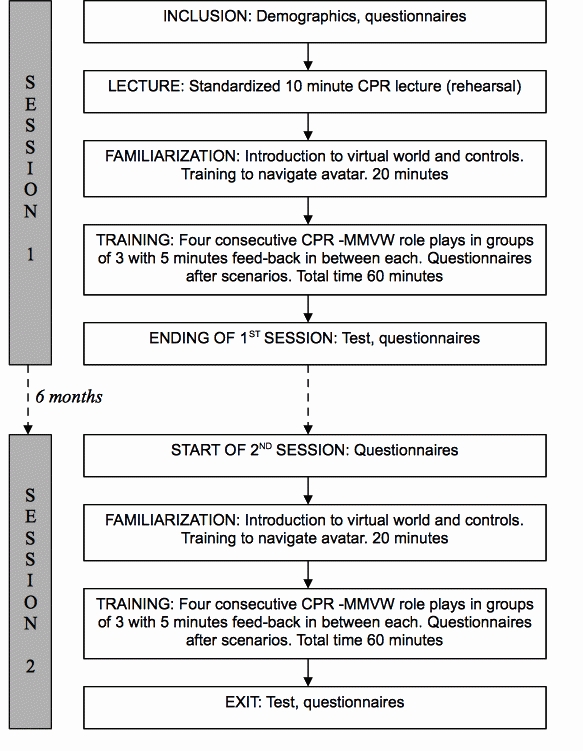
Design of the study in a test-retest manner

A previously developed virtual world (Forterra Systems Inc, OLIVE, game development platform) with prehospital CPR training capabilities was implemented. In mixed groups of three, the subjects attended two structured two-hour sessions six months (185-204 days) apart. Figure 1 summarizes the content and extent of the training sessions.

A short 10-minute lecture on adult basic life support adhered to 2005 guidelines from the European Resuscitation Council [27]. Besides focusing on when and how bystander CPR should be performed, other life support areas—precautions when performing out-of-hospital CPR and foreign body airway obstruction—were briefly covered. This presentation mainly served as a theoretical review of content covered during the previous conventional training. However, newer 2005 guidelines had, during the time that had elapsed, been fully implemented in Sweden. In the subsequent familiarization period, the subjects were acquainted with the interface features of the virtual world simulation. The interactions in the virtual world were controlled by the subjects using the computer keyboard, mouse, and headset. The subjects´ engagement modes were assessed before starting the scenarios using a validated 15-item questionnaire from which values for positive and negative engagement modes were calculated. This has previously been described in detail by Hedman et al [28]. In order to also control for different individual coping strategies during the two sessions, we administered a validated questionnaire described by Carver [29] before the scenarios, in which each of 21 items gave indications of a particular way of coping.

**Figure 2:**
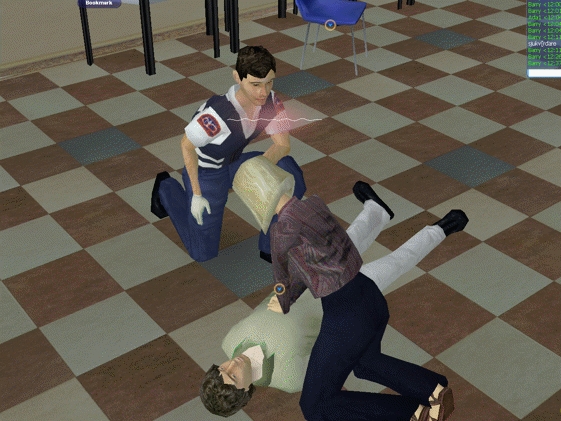
Screenshot from scenario inside classroom

In the virtual world simulation, the subjects interacted in real time with each other, a victim, and paramedics, by use of their avatars. The trainees were instructed to approach a victim who had collapsed in the virtual world, take the correct diagnostic steps, and collaboratively perform the cognitive and procedural measures associated with bystander CPR. These included (1) moving to the victim, (2) checking the victim for consciousness, (3) declaring the victim unconscious, (4) checking the victim’s airway and breathing, (5) calling for help, (6) performing chest compressions and rescue breaths as stated by the CPR protocol, (7) relieving the rescuers, and (8) assisting the arriving paramedic and giving a brief report to him. Each group consisted of 3 medical students trained in 4 role plays (ie, scenarios). The first two scenarios occurred in a classroom followed by two scenarios that occurred outdoors in a parking lot. A screenshot from the classroom scenario is shown in Figure 2. The scenarios ended when a paramedic entered the virtual world and assisted the group. After each scenario, the trainees received classroom feedback from an instructor. The feedback focused on the bystander CPR protocol and how the actions performed in the scenario complied with the protocol. In general, the feedback sessions took 5 minutes or less.

During the second session held six months later, the subjects received the same curriculum without the lecture. The virtual world scenarios were the same as in the first session. No subjects dropped out between the training sessions.

Demographic data of the study subjects are displayed in Table 1. The values shown are based on self-report at inclusion in the study. There were no significant differences between males and females. 

**Table 1:** Demographics

	Total (n = 12)	Male (n = 6)	Female (n = 6)
Age, mean (SD)	22.6 (3.5)	23.2 (4.8)	22.0 (1.7)
Number with access to computer at home	12	6	6
Level of computer experience (0 to 3)^a^ median (range)	2 (1-2)	2 (1-2)	2 (1-2)
Computer and video game use (0 to 5)^b^ median (range)	1 (0-4)	3 (0-4)	1 (0-2)

^a^ Level of computer experience was graded on a 0 to 3 Likert-type scale where 0 = none and 3 = very high.

^b^ The use of computer and video games was graded on a 0 to 5 Likert-type scale where 0 = none, 1 = less than once a month, 2 = once every second week, 3 = once a week, 4 = several times every week, and 5 = every day.

In order to get information about the qualitative experiences from the virtual world training, a questionnaire was administered at the end of each session. The subjects were asked if they found the virtual world simulation to be realistic, easy to use, and useful in preparing them for future tasks. Subjects were asked to rank their answers on 5-grade Likert type scales where 1 = not at all and 5 = very much. Further, the subjects were asked to state the strengths and weaknesses of the training scenarios. The questions were open-ended, and the subjects could list as many examples as they wished. Lastly, the subjects were asked if they had learned anything new. Answers to this question were given as written free comments by the subject. These were then fragmentized into meaning-bearing entities, analyzed, classified, and categorized by the authors. If the same kind of answer was given several times by one subject, it was counted as only one occurrence to have it justly weighted.

Knowledge about the material covered by the lecture—adult basic life support (BLS)—was measured by a written examination using a 10-item questionnaire that was administered at the end of each session. Each question was followed by four true-or-false statements. The answer was considered correct if all four statements were correctly answered.

Perceived self-efficacy [20], as one affective component of learning [21], was self-assessed before and after each training session using a 5-item questionnaire where each item was rated on a 7-grade Likert-type scale. Self-efficacy was then calculated as the mean value of these items.

Concentration is a skill to selectively focus on relevant information while ignoring distractions and can be associated with this type of training [24,25]. Intense concentration is highly associated with the “flow experience”, defined as an internal state of total focus, involvement, and absorption in an ongoing task [22]. We used eight familiar items on concentration from previously validated instruments for assessing flow experience [22,28,30,31]. Concentration was measured immediately after the first, second, and forth scenario using a 0 to 100 visual analogue scale, and the concentration score was calculated as the mean of all items.

The mental strain during the training was measured by one question directly after the first, second, and forth scenario using a validated instrument, the Borg´s CR10 scale, in which subjects were asked to rate their mental strain on a scale of 0 to 10, where 0 = no mental strain at all and 10 = extremely high mental strain [23].

Repeated measurements analysis was used to analyze time dependent data, and regression analysis was used to evaluate the dependency between variables. Statistical comparisons to test differences between two groups were made by use of the Mann-Whitney U test or by use of the Student’s *t* test for uncorrelated means after validation for normal distribution by use of the Shapiro Wilk test. The within group analysis was made by use of pairwise Student’s *t* test for correlated means. Multiple comparisons of continuous data were performed by analysis of variance. The procedure proposed by Fisher was used to control for multiplicity [32,33]. In order to evaluate hypotheses of variables in contingency tables, the chi-square test was used or, in the case of small expected frequencies, Fisher's exact test. In addition, descriptive statistics and graphical methods were used to characterize the data. The study employed multiple hypotheses testing where each hypothesis was analyzed separately and the existence of patterns in and the consistency of the results were considered in the analysis. All analyses were carried out by use of SAS, version 9.1 (SAS Institute, Inc, Cary, NC, USA), and the 5% level of significance was considered. Data are presented as means (SD) or medians (range), depending on the type of distribution.

## Results

### Subjective Reflections

Results of subjects’ scoring of realism, ease of use, and usefulness in future work are presented in Table 2.

The first two questions were asked after both sessions, but there were no significant changes and the data are presented as mean values of both sessions. The questions on usefulness were only asked after session 2.

The subjects’ answers to the question pertaining to strengths and weaknesses of the simulated scenarios are summarized and presented in Table 3.

**Table 2:** Subjects’ scoring on realism, ease of use and usefulness in future work

Question^a^	Female (n = 6) mean (SD)	Male (n = 6) mean (SD)
Did you find the simulations realistic?^b,d^	3.92 (0.29)	3.04 (0.75)
Did you find the computer program easy to use?^b^	3.88 (0.74)	4.17 (0.58)
How useful do you think these exercises would be for learning to react to a medical emergency?^c^	4.50 (0.55)	4.17 (0.41)
How useful do you think these simulation exercises would be for learning to work together as members of a health care team?^c^	4.67 (0.52)	4.00 (0.63)

^a^ Scoring performed on 1 to 5 grade Likert-type scale where 1 = not at all and 5 = very much.

^b^ Question asked after both sessions. Mean values are combined scores for both sessions. No significant changes were found over time

^c^ Question asked only after session 2. See text for details.

^d^ Difference between males and females statistically significant (P < .05, *t* *22* = 3.76).

**Table 3:** The subjects’ answers to the question pertaining to strengths and weaknesses of the simulated scenarios.

Answer Categories		Number of Statements in Category
**Strengths**		
	Suitable and realistic environment	14
	Good way to repeatedly practice	9
	Necessary to adapt to changing circumstances	7
	Easy and straightforward	4
	Training teamwork aspects	2
	Learning about own reactions	1
**Weaknesses**		
	Tasks too easy, more options wanted	23
	Lack of realism and environment not rich enough	9
	Technical problems	2

In response to the question, “Have you learned anything new,” 11 subjects said yes and one subject said no. When asked in which field new learning had occurred, 8 of 12 subjects responded that new learning was mainly attributable to a newer version of CPR guidelines, 4 of 12 responded they had learned more about teamwork, and 4 of 12 said they had learned more about working under stress. When discussing how scenario-based team training in the virtual world was perceived, a typical comment was, ”I think that it is a good way of learning, which can attract a large group of experienced computer users more than other CPR education (with a manikin, although the manikin is needed to get the practical aspect).” Another reoccurring comment was a request for more “situations (with greater variation), preferably [with] people around, which increases stress and sense of reality.”

### Other Individual Experiences and Strategies

**Figure 3:**
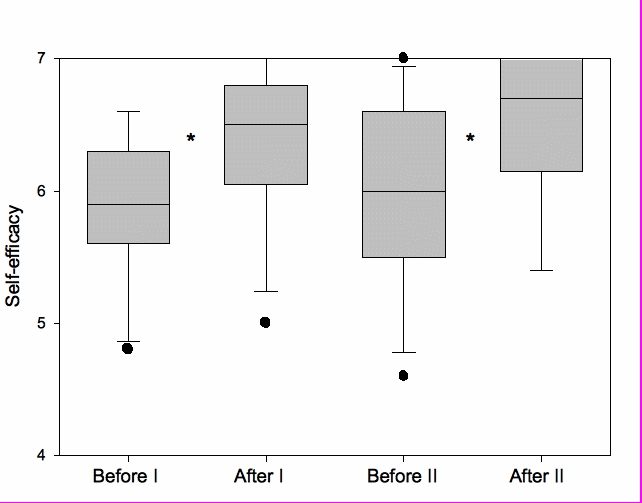
Self-efficacy in the study group

Engagement modes and coping strategies were stable over time (data not shown). In Figure 3, box-plots illustrate self-efficacy at baseline and after training.

**Figure 4:**
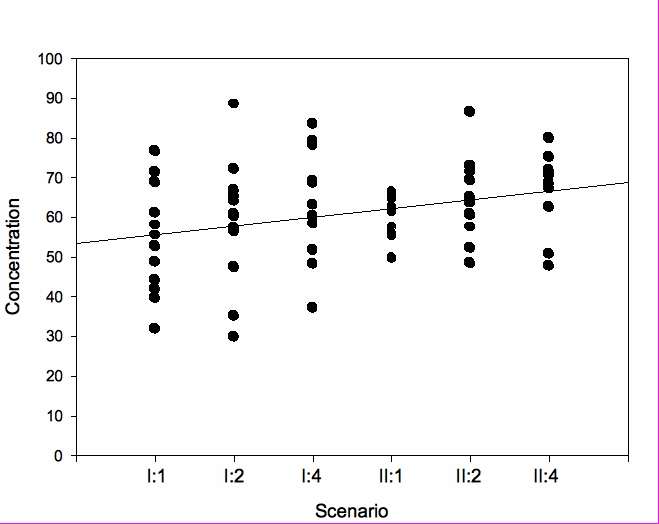
Scatter plot depicting the individual score for concentration at each scenario

A significant increase was observed in self-efficacy for the whole group after the first session and also after the second session. Further, there was a statistically significant difference in self-efficacy between males and females before the training. The mean (SD) self-efficacy for female subjects was 5.47 (0.47); the corresponding value for male subjects was 6.20 (0.40) *(P =* .015). Thereafter, no difference in self-efficacy was observed between males and females. The change of concentration over time is shown in Figure 4.

In the first session, the mean (SD) concentration value was 54.2 (13.7) during the first scenario. During the second scenario, it had increased to 58.7 (15.8), and during the fourth scenario, it reached 63.1 (15.6). In the second session, the corresponding values were 59.3 (5.1), 65.1 (10.4), and 66.6 (9.4) respectively (*P* = .006). For female subjects, mean (SD) concentration increased from 60.1 (10.6) to 71.4 (4.4), whereas for men, the increase was from 48.3 (14.8) to 61.8 (10.9). No significant changes in mental strain were noted over time either in the complete group or in any of the subgroups. However, over all scenarios, the mean (SD) mental strain value for men was 1.2 (0.7), significantly lower than for women whose mean (SD) mental strain value of all scenarios was 3.7 (1.7) (*P* < .001). No correlation between mental strain and gaming experience was found.

### Knowledge

The mean (SD) results on the written examination was 8.0/10 (1.6) after the first session and 6.25/10 (1.5) after the second session. This 20 percent drop was statistically significant (*P* = .002).

## Discussion

As, to our knowledge, very few examples of virtual world-based team training using avatars exist in the medical field, our objective was to understand how the use of this tool for CPR training was perceived by medical students. Despite leisure gaming being common and popular, it is debatable whether it can be useful in the field of serious games. An important finding was that the subjects, in general, felt positive about the virtual world training experience. Although all subjects had previously participated in traditional CPR training, a majority learned something new, and all were positive toward further virtual world training. Interestingly, the subjects rated the future potential and usefulness of this type of training highly. Although many of these findings must be regarded as context specific, it appeared that the major benefits from this type of training were in its usefulness to practice repeatedly in a scenario-based setting. The subjects found the training quite realistic, easy, and straightforward. Contrary to conventional CPR training curriculum, team aspects, probably important for real-life outcome, were introduced in this training. Also these measures seemed to be appreciated by the trainees. The weaknesses with the virtual world training were mainly related to the virtual environment itself; realism, increased diversity among the scenarios, and more challenging medical cases were asked for. Although there were several occurrences of technical imperfections, we found comments from only two subjects regarding technical problems.

The relatively high scores of self-efficacy found before the first training session, partly explained by recent conventional CPR training, increased to even higher scores after the training. After 6 months, the self-efficacy scores showed a numeric decrease that did not, however, reach statistical significance. After the second session, the scores increased again. We interpret this as an indication that virtual world scenario-based team training can be used effectively to rebuild confidence, if not competence. Despite the small sample size, it is important to note the differences between female and male subjects. The ratings for the female subgroup were in this aspect inferior, indicating less confidence, compared with those of the male subgroup during the whole study period.

Previous research has shown that effective training is correlated with a high degree of concentration [24,25]. During the first session, subjects’ concentration was on a medium level and increased over time. This trend was also replicated in the second session. The reason for this could be due to subjects’ clearer understanding of the tasks, their better control over how to maneuver in the virtual world, as well as their having fewer technical problems. However, increased ability to concentrate might also come from learning to work as a team, that is, as teamwork becomes smoother and the resuscitation process is perceived as more effective and rewarding, concentration increases. In the exit questionnaire, we noted that generally the scenarios were considered quite easy. However, it didn’t appear as if the perceived easiness of the scenarios had any detrimental effects on concentration, although if the scenarios were better matched to expectations, it could be hypothesized that scores on concentration in general would be even higher. The higher score on concentration for the female subgroup might indicate a higher degree of involvement of females in these scenarios.

The experienced mental strain was in the medium or low range of the test scale. Mental strain can certainly be modified by the trainer, the expectations, and the setting of the training. High scores might be typical for the real world CPR situation, but generally too much mental strain might hamper learning. Interestingly, even with moderate mental strain, several subjects found the training to be useful in learning about their own reactions to stress. The difference between male and female subjects was obvious and persistent over time, indicating higher involvement in the task among the female subgroup during the scenarios. Although the level of previous gaming experience seemed to be higher in the male group, it is less likely that this fact contributed to the higher mental strain in the female subgroup, since no correlation between gaming experience and mental strain was found. Also, women in this study did not experience technical difficulties to a greater extent than men.

One intention in this study was to assess whether situated learning occurred. Therefore, the theoretical lecture on CPR allowed a common level of knowledge among the subjects to be set. The lecture was omitted in the second session. If situated learning did occur to any greater degree, we would expect the subjects to activate and retain this knowledge merely by repeated practice. We observed a significant decrease in knowledge of BLS between the test and retest. Assessing knowledge retention was possible by using the same quiz about information provided in a theoretical lecture. What this drop of knowledge actually reflected is debatable; the content of BLS is somewhat broader than bystander CPR, therefore, some aspects were not practiced in the virtual world. Hence, it could be argued that part of the knowledge that was tested was not actively sustained by training. One could also argue that this observation could reflect the lack of haptic chest compressions in MMVW simulation. Adding psychomotor activity, that is, simulated chest compressions, might increase the fidelity and enhance learning as well as recall of knowledge. In Sweden and many other countries, the established procedure of the resuscitation community is that annual training is warranted for professionals, and biannual training or less frequently for nonprofessionals. These long intervals may account for the poor efficacy of current training methods [34,35]. A potential solution may be to increase the sense of presence by adding a CPR manikin to the MMVW for a more engaging and integrated opportunity to practice and establish the routine.

These are clearly important aspects that are not specifically addressed in current CPR education and training. The reactions from the students also support further development of this tool as many in this group asked for more training options, technical possibilities, and different medical scenarios. Finally, the observation of lower confidence, reduced sense of self-efficacy, higher levels of concentration, and the greater mental strain experienced by females suggest the need for further studies of how different students learn resuscitation with this method. Further systematic studies with control groups are needed to understand the main reasons for such differences, that is, whether these are biological, cultural, or adaptive. We are unaware that these individual experiences have been evaluated with current manikin-based training methods. In order to better understand the nature of the experiences of trainees when using MMVW to learn CPR techniques, it would also be interesting to focus in depth on trainees’ reactions and reflections. Another important area to be covered by future studies is how the trainers and stakeholders react to virtual world training. If this type of training is to be successfully implemented, it is of paramount importance that these groups find value in such alternative training methods.

Previously identified weaknesses in current CPR practice include stress, time constraints, lack of nontechnical skills among team members, uncertainty over the need to act, risk of inflicting harm, and lack of procedural competence. Virtual world training can address the majority of these problems. Most obviously, MMVW can be used to train the procedural competence, but the strictly physical aspects (eg, how to perform rescue breaths and chest compressions) cannot be trained with the current method. Hence, this type of virtual world training cannot stand alone to train people in CPR. Instead, it can be used to help students gain cognitive and team training skills, and therefore it can work as an adjunct to other manikin-based training.

Engagement modes and coping strategies were stable over time, and probably did not affect the students´ performance and other subjective reactions differently during the sessions.

To summarize, scenario-based virtual world team training of CPR using avatars seems to be engaging and elicits positive changes in students´ subjective experiences. The results demonstrate the potential of serious gaming technology as a new tool in medical education and training. Once the problem of the developmental costs of software has been solved, virtual worlds for training for medical emergencies might be easy to disseminate and be engaging, as well as enable new teaching strategies and pedagogic development.

### Limitations

An obvious limitation of this study is its small sample size. However, the purpose was to perform a small exploratory study with a new training tool in an established learning context (medical school). The training effects might have been sensitive to factors other than training, such as technical difficulties, attitudes in the whole group towards this type of scenario-based simulator training, and previous computer and video game experiences. One way to partly control for this type of bias in the present study was the inclusion of the self-efficacy instrument and questions in the exit questionnaire. Our choice of the 6-month interval may have exceeded the capability of the subjects to recall accurately the details of CPR performance; shorter intervals may produce more favorable results.

### Conclusions

Cardiopulmonary resuscitation is a critical competence that is necessary to disseminate in society in order to save lives. With a new emerging generation of health care staff accustomed to gaming technology since childhood, serious games for education and training have a great potential. Although more evaluations on the effectiveness and customizing of this technology are necessary, we have found supporting evidence for scenario-based virtual world training to be used to supplement and reinforce traditional education and training, in particular, to prolong retention of knowledge and procedural order. MMVW training using avatars offers several advantages since it is easily distributed, requires no specialized equipment, and may be carried out as distance training.

## Abbreviations

BLS: basic life support
CPR: cardiopulmonary resuscitation
MMVW: massively multiplayer virtual world
SUMMIT: Stanford University Medical Media and Information Technology
